# Surface analysis of ureteral stent before and after implantation in the bodies of child patients

**DOI:** 10.1007/s00240-020-01211-9

**Published:** 2020-09-09

**Authors:** Katarzyna Arkusz, Kamila Pasik, Andrzej Halinski, Adam Halinski

**Affiliations:** 1 Department of Biomedical Engineering, Faculty of Mechanical Engineering, University of Zielona Gora, 9 Licealna Street, 65-417 Zielona Gora, Poland; 2 Department of Paediatric Urology, Cherry Clinic, Anieli Krzywon 2 Street, 65-534 Zielona Gora, Poland

**Keywords:** Ureterorenoscopic-lithotripsy procedure, Ureteral stent, Kidney stone, Scanning electron microscopy, Mechanical strength

## Abstract

The aim of this work was to determine which part of a double-J ureteral stent (DJ stents) showed the highest tendency to crystal, calculi, and biofilm deposition after ureterorenoscopic-lithotripsy procedure (URS-L) to treat calcium oxalate stones. Additionally, the mechanical strength and the stiffness of DJ stents were evaluated before and after exposure to urine. Obtained results indicated that the proximal (renal pelvis) and distal (urinary bladder) part is the most susceptible for post-URS-L fragments and urea salt deposition. Both, the outer and inner surfaces of the DJ ureteral stents were completely covered even after 7 days of implantation. Encrustation of DJ stents during a 31-day period results in reducing the Young’s modulus by 27–30%, which confirms the loss of DJ stent elasticity and increased probability of cracks or interruption. Performed analysis pointed to the need to use an antibacterial coating in the above-mentioned part of the ureteral stent to prolong its usage time and to prevent urinary tract infection.

## Introduction

Nephrolithiasis is a frequent cause of morbidity (12%) in the world population, and half of those affected will have recurrent stone disease [1]. Previous research on urinary stones considered only adult patients, completely omitting the paediatric population, whereas there is mounting evidence for increased stone formation in younger patients [2]. Shock wave lithotripsy is in majority of the cases the first-line treatment method to eliminate the kidney stones. In case of ureteral stones, especially in the lower part of the ureter ureterorenoscopic-lithotrypsy (URS-L) procedure might be performed. After surgery, the implantation of a double-J ureteral stent may be needed. There are absolute indications for stent insertion, which include relief of obstructed pyelonephritis, bilateral ureteral obstruction, ureteric injuries, and post-treatment of urolithiasis in patients with a solitary kidney [3, 4]. As no ideal stent, many problems of stent migration, occlusion, encrustation, fragmentation, and stone formation were noticed.

The materials used for the production of ureteral stents should comply with a number of specifications. Materials required for urinal catheterization should have a smooth surface (low surface roughness) [5], high mechanical strength, and flexibility [6], and they should be biocompatible, antimicrobial, and antifouling (being able to prevent microbial accumulation or to interfere with the biofilm structure) [7]. Furthermore, these materials should be able to resist bacterial adherence and subsequent biofilm formation [8]. The most common materials used as a ureteral stent are polyethylene, polyurethane, and silicone. Rigidity and tendency to break are the serious problems in using these materials in clinical setting, which presents a variable tendency to encrustation. Among these materials, the silicone surface is smoother than the others, and has the best performance in the long term, showing 30% less encrustation at 10 weeks, and indicates low susceptibility to calcium deposition [9]. On the other hand, there can be differences in the composition of encrustation at each end of a stent [10]. The rate of encrustation was 18.33% in the first 5 weeks, 56% between week 6 and 12, and 75% thereafter [11]. Additionally, the calculi or crystal deposition promoted the biofilm formation, which affected the urinary tract infection.

Adhesion is a key determinant, which initiates every stage of the pathogenesis of urinary tract infection [12]. The main process of biofilm formation is binding bacteria to a ureteral stent. The type of materials used in the ureteral stent has a significant impact on microbial cell adhesion and the rate of bacterial biofilm development [7, 13–15]. The biofilm forming on the surface of the ureteral stent may form the core for urinary stones. The aggregation of the biofilm produced by the bacteria and the precipitated urinary components cause the formation of kidney stones on the surface of ureteral stents [16, 17]. Stents become blocked due to encrustation caused by the formation of these structures [18]. To reduce the adhesion of microbial cells and the rate of bacterial biofilm development, urological catheters are coated with different substances, such as nano-silver [7, 19, 20], metallic nanoparticles [20, 21], Lysostaphin [22], Chlorhexidine [23], antifouling zwitterionic moieties (containing an equal number of positively and negatively charged functional groups) [24], polyvinylpyrrolidone (PVP, a water-soluble polymer) [8], nitrofurazone [19], polytetrafluoroethylene (PTFE), hydrogel [5], and impregnation with a combination of rifampicin, sparfloxacin, and triclosan [25]. Bacterial biofilm formation and encrustation may cause obstruction or blockage of ureteral stents [8].

Limited work has been conducted to carry out the mechanical characteristic of the ureteral stents before and after exposure to artificial urine (30 days) in static conditions [26]. Only a few studies have been performed to evaluate the effect of a stent’s rigidity on patients’ quality of life [27–29]. No correlation between the material composition and the patients’ quality of life was observed [27]; however, the stiffness of the ureteral stent was associated with higher incidences of dysuria and pain [28, 29]. Hitherto, studies of ureteral stents included determination of effective urine flow from the kidney to the bladder [30], investigation of structural and chemical composition [11], and shape and size optimization [31]. The urine flow consists of in-stent (luminal) and out-of-stent (extra luminal) flows, whereby lower flow rates were observed with larger DJ stents, and increasing the number of side holes increased the overall flow rate [30].

Trends in research on the ureteral stent focus on the development of effective methods of combating biofilm, preventing kidney stone formation, and improving the patients’ quality of life. Mainly, these trends come down to impregnating the ureteral stent with antimicrobial substances (antibiotics or antiseptics) [32]. However, in the literature, there is no definite answer concerning the effectiveness of these layers in the elimination of infection or obstruction of microbial colonization of the catheter wall in comparison to uncoated stents. Therefore, there is a need to study not only the mechanism of formation of kidney stones or biofilm on the catheter surface and methods to prevent these processes, but also to study the influence of these structures on the properties of ureteral stent. This paper focuses on determining which material factors show the highest affinity for the ureteral stent surface and the distribution of these components along the entire length of the stents. Moreover, the effect of the time of implantation in child patients on kidney stone formation in both brand new and used catheters was evaluated.

## Materials and methods

### Materials

A total of 15 double-J ureteral stents (DJ stents) were placed during 15 procedures in the 15 children patients. The ureteral stents were built with a proprietary silicone-modified styrene/ethylene/butylene block copolymer. The mean length of the double-J ureteral stents was 20 ± 2 cm (range 8–32 cm). The mean placement duration was 17.25 ± 10.21 days (range 7–31 days).

### Microscopic analysis of stents

An analysis using scanning electron microscopy with energy-dispersive X-ray spectroscopy (SEM/EDS) was performed on the brand new ureteral stents as the reference analysis, as well as on the separate ureteral stents implanted after the ureterorenoscopic-lithotrypsy (URS-L) to treat calcium oxalate stone. Additional energy-dispersive X-ray spectroscopy (EDS) analyses provided information on the elemental composition of every sample.

The microscopic analysis was performed by the scanning electron field-emission microscope JEOL JSM 7600F equipped with an X-ray analyser INCA OXFORD according to the previously elaborated procedure [14, 15]. The microscopic observation of the ureteral stents required the use of an additional sample preparation procedure which includes: the immersing of samples in a 25% solution of glutaraldehyde in a phosphate buffer ($\hbox {pH}\,7.2$) and rinsing (3 times) in a deionized water at room temperature. Samples were then dehydrated in 10 ml portions of acetone–water solutions with concentration rising from 10 to 100%. Drying was carried out at the critical point of CO$_{2}$, using the critical point E3000/E3100 drying apparatus (CPD). The ureteral stent surface was covered with a chromium layer with the thickness of 5 nm. The glutaraldehyde and acetone were purchased from Sigma-Aldrich.

### Determination of mechanical properties

Tensile strength or stiffness was tested for all double-J ureteral stents before and after urinary exposure. Each DJ stent had 3–5 stents tested. The tensile strength was determined using an MTS Micro Bionix Testing System (Laboratory of Prototyping of Medical Devices, Department of Biomedical Engineering, University of Zielona Gora) equipped with Testworks II software, vibration isolation table, and grips, using an 5 N load cell. Uniaxial tension was applied to each stent with a testing rate of 1 mm/s for 1 s. A preconditioning run was done for each stent, including a 3-min hold time at 5 mm with 30 s between the preconditioning run and the first trial. Force was reduced to 0 between each trial. Each DJ stent (brand new and removed from the patient’s body) was examined using the tensile test minimum times, keeping the same manner of reposition. To determine the impact of the above conditions on susceptibility to damage to the stent, mechanical characteristics tests were used through determining the parameters: Young’s modulus, i.e., the elasticity of the material (the greater the Young’s modulus, the more elastic the material is and the lower the risk of its damage) and ultimate force, i.e., the maximum force that must be applied to the stent to tear it apart (the lower the strength, the more fragile the stent is).

## Results and discussion

### Microscopic analysis of the surface of ureteral stents

**Fig. 1:**
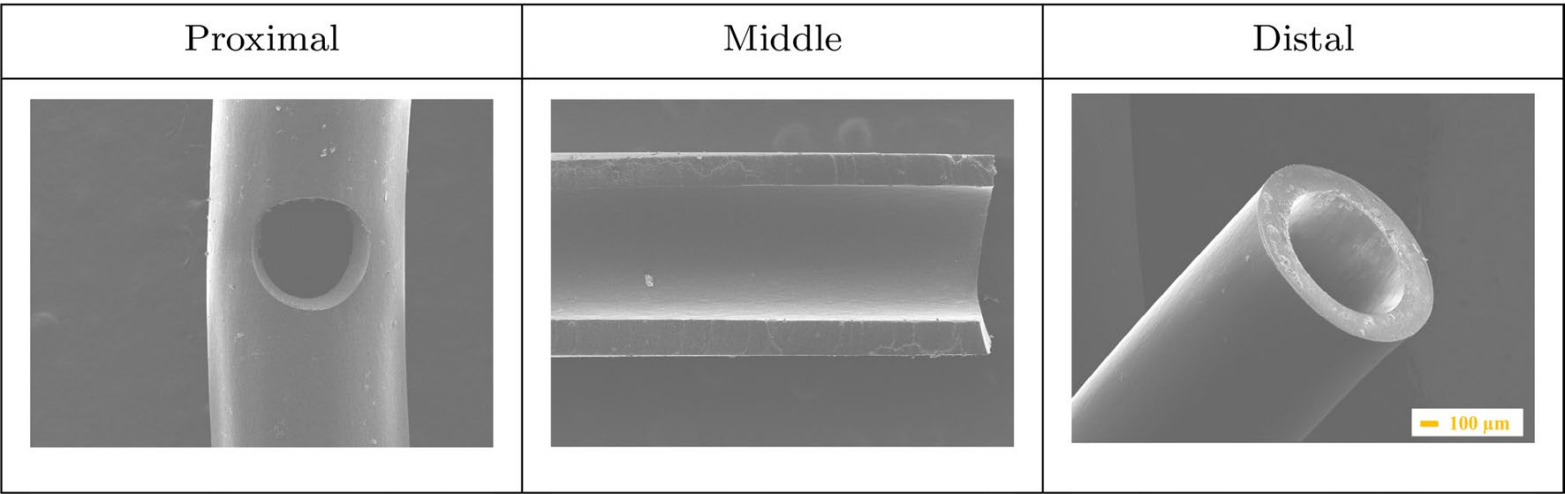
SEM micrographs of brand new double-J ureteral stents

**Fig. 2:**
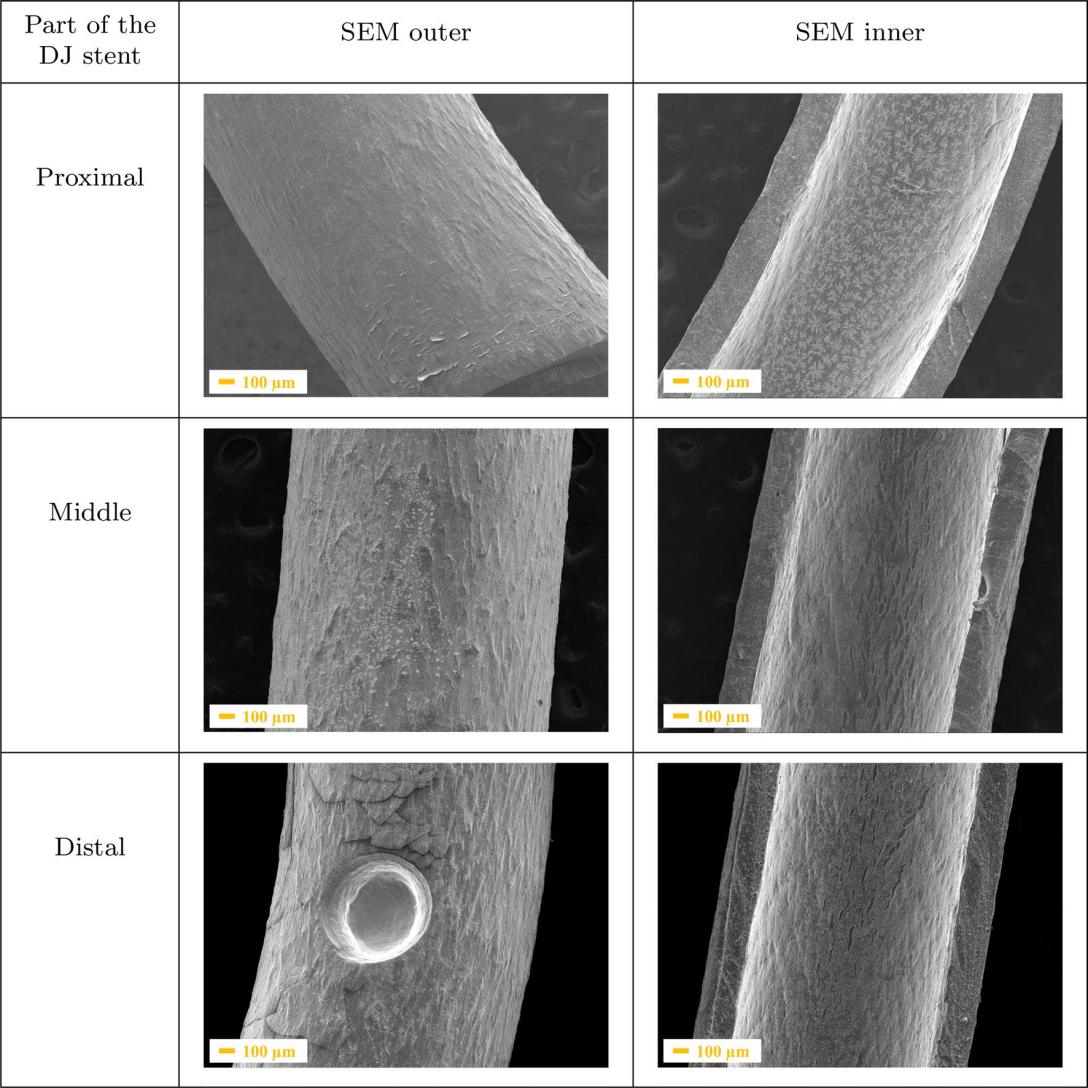
SEM images of a ureteral stent implanted during 7 days, where: **a** is the proximal, **b** is the middle, and **c** is the distal part of the stent

**Fig. 3:**
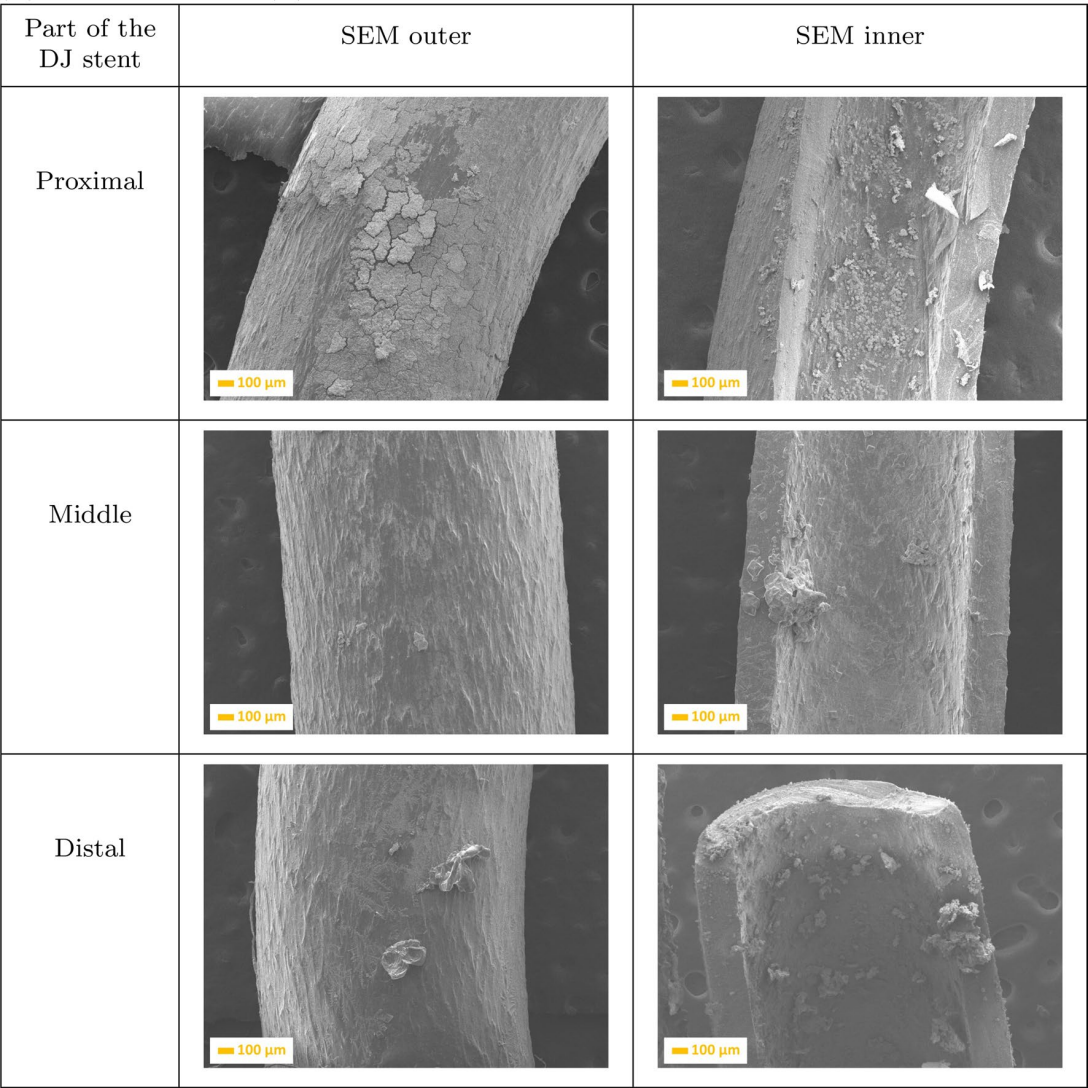
SEM images of a ureteral stent implanted during 13 days, where: **a** is the proximal, **b** is the middle, and **c** is the distal parts of the stent

**Fig. 4:**
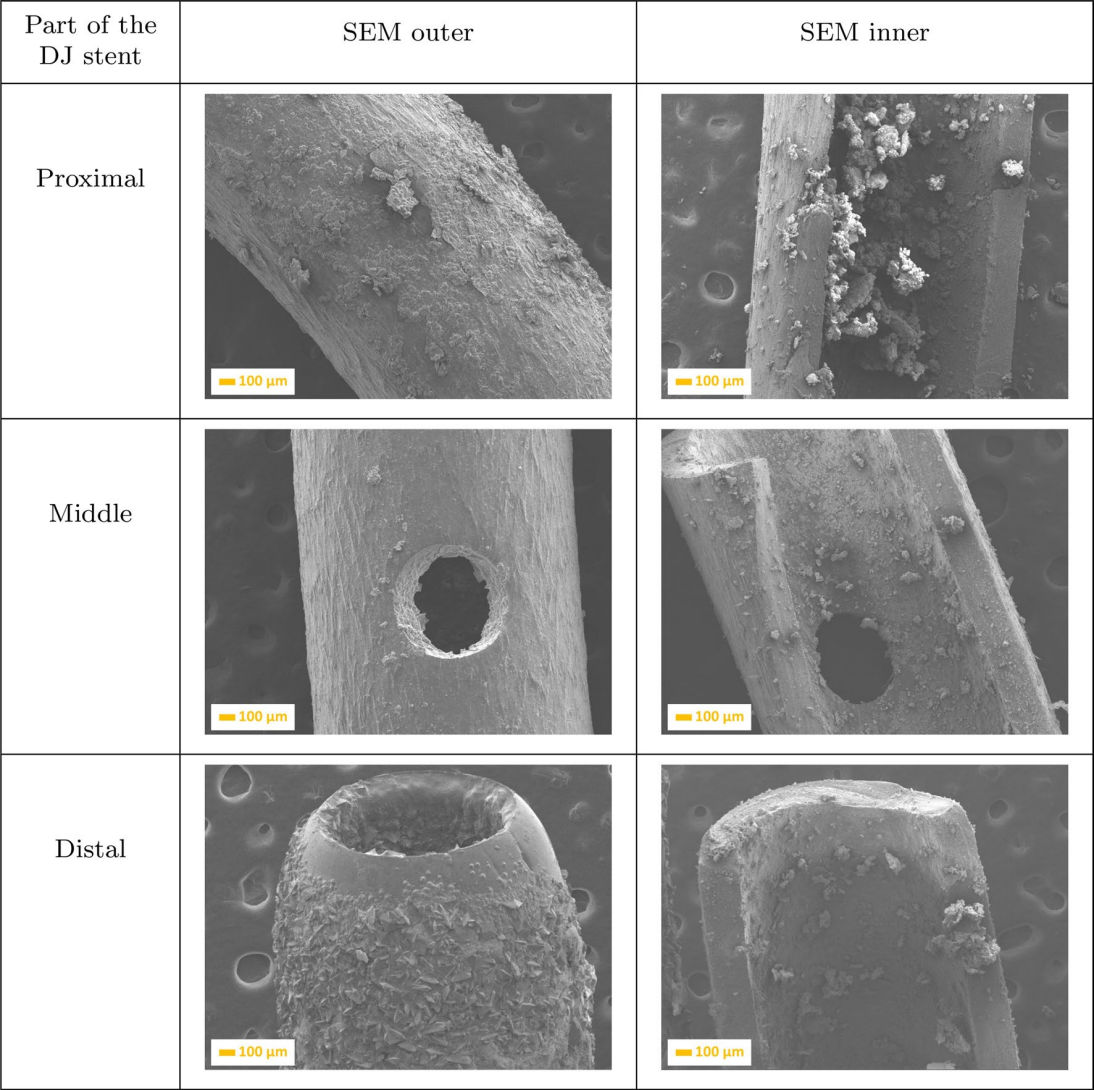
SEM images of a ureteral stent implanted during 18 days, where: **a** is the proximal, **b** is the middle, and **c** is the distal parts of the stent

**Fig. 5:**
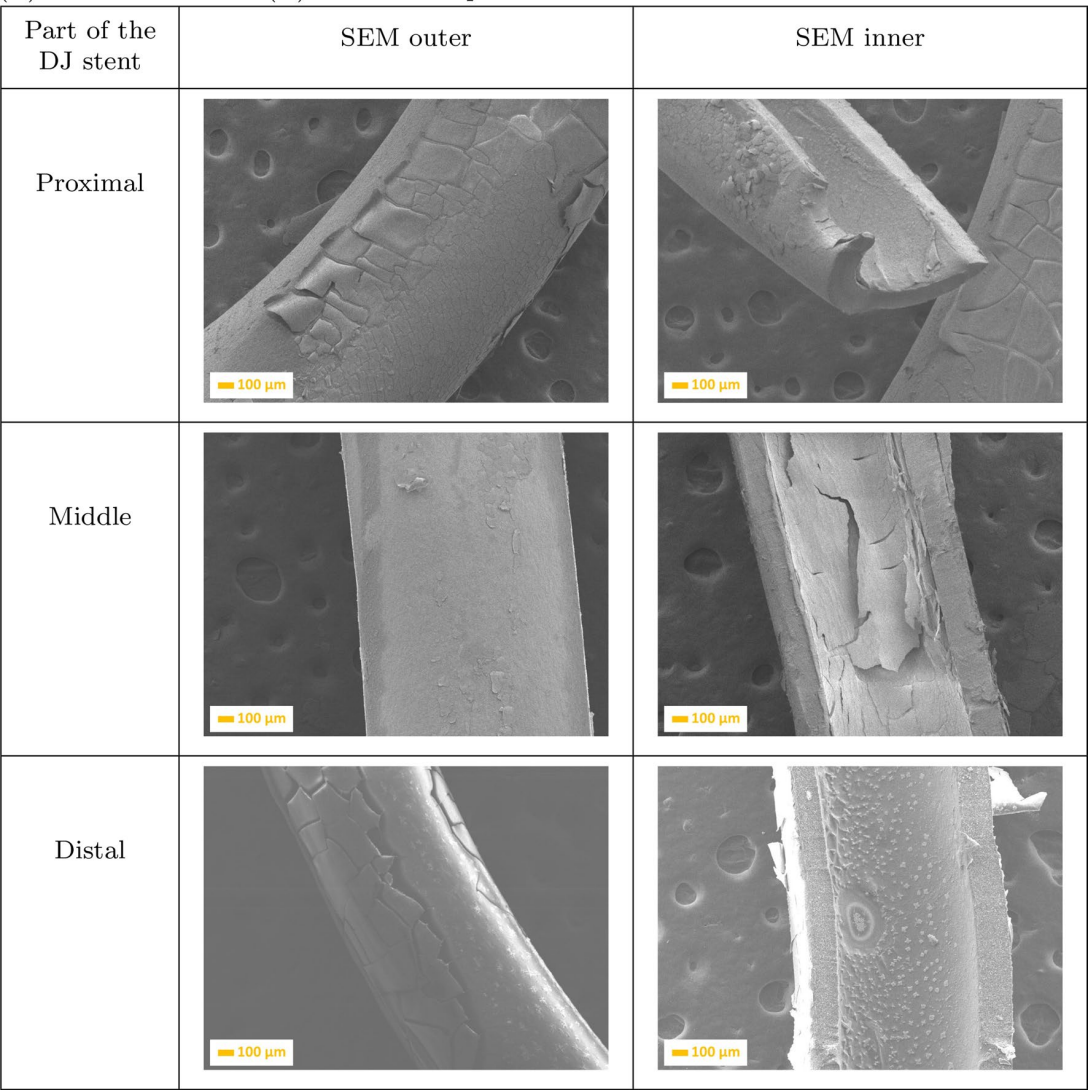
SEM images of a ureteral stent implanted during 31 days, where: **a** is the proximal, **b** is the middle, and **c** is the distal parts of the stent

The surface morphologies of double-J ureteral stents (DJ stents), with identical shapes and number of side holes, have been investigated using scanning electron microscopy and are shown in Figs. 1, 2, 3, 4 and 5. The structure of brand new DJ stents with markings used in the text is shown in Fig. 1, where the proximal part of double-J ureteral stents means “pigtail”, which is situated in the renal pelvis, while the distal coil is situated in the urinary bladder.

A separate batch of double-J ureteral stents was implanted after ureterorenoscopic-lithotrypsy to treat calcium oxalate. The period of implantation in child patients’ bodies was 7, 13, 18, and 31 days, respectively. As can be seen in Figs. 2, 3, 4 and 5, the kidney stone was deposited on the outer and inner surfaces of each analysed ureteral stent. Representative scanning electron micrographs of a ureteral stent (Fig. 2) confirmed the encrustations and biofilm formation after post-URS-L implantation for 7 days. SEM imaging indicated thick crystal-containing layers in distal section of the DJ stents, confirmed in the literature as the weddellite, frequently in a tetragonal dipyramidal shape (calcium oxalate dihydrate, $\mathrm{CaC}_{2}\mathrm{O}_{4}\cdot 2\mathrm{H}_{2}\mathrm{O}$) [33]. Moreover, the side hole located in the distal section of the DJ stents was completely filled by calcium oxalate film. Each site of the middle part of the DJ stent was covered by a thin, compact layer of post-URS-L encrustation. The proximal inner part of the DJ stent showed NaCl dendrites starting and growing from the surface irregularities. The dendritic growth is the first stage of NaCl crystal formation. When urea reaches the dendrite, it forms a square crystallite whose size depends on the size of the liquid blob. Crystal growth is initiated in their corners causing their further linear attachment at a right angle. This network of branches with an intricate rectangular pattern has been shown to be multi-fractal [34]. The most extensive agglomeration observed during the seventh day of implantation could be the result of cleaning the kidney of post-URS-L residues. This is a crucial moment in blockage of the DJ stents. If the diameter of the DJ stent is to large, the flow rate is too low, which extends the contact time of post-URS-L fragments with the surface of the DJ stent, resulting in its blockage.

After 13 day implantation of the double-J ureteral stent (Fig. 3), the post-URS-L fragments were deposited in each fragment of the inner part of the stents. However, the proximal part showed greater affinity. It can be observed that the outer surface was more covered by the crystals, which form the compact layer that cracks during sample preparation. If the diameter of the DJ stent has been chosen correctly, the extension of implantation time results in a continuous increasing of the thickness of the multi-layer on the inner side of the proximal part of the stent.

Extension of implantation time to 18 days (Fig. 3) resulted in increasing the amount of crystals (fragments of kidney stones) in the proximal inner part of the double-J ureteral stent, almost closing it. It can been observed that crystals of calcium oxalate are located primarily in the inner side, and embedded also on the holes in the middle of the DJ stent. The outer, distal part was completely covered by a uniform layer of kidney stone fragments, which formed a multi-layered structure.

Analysis of the DJ stent implanted during 31 days (Fig. 4) confirmed that the deposition of calcium oxalate formed a uniform layer on the whole surface of the ureteral stent, on both the inner and outer sides. Extension of implantation time resulted in the formation of a compact layer, leading to pain and a difficult removal. After 31-day implantation, it can be seen that the outer (Fig. 5) side is completely covered by small calculi, growing on one another.

Among the clinical problems related to using the double-J ureteral stent, major complications, such as encrustation, vesicoureterical reflux, urinary infection, migration, stent fracture, ureteroarterial fistula, and necrosis, are distinguished [36]. Furthermore, the ureteral stent patency, defined as seeing flow from the distal end of the stent in the urinary bladder or iodinated contrast in the renal pelvis, is difficult to assess [37]. Hitherto, the investigation on flow and encrustation of ureteral stents pointed out that long-term stent use is associated with infection and precipitation of salts from the urine, which can lead to a build-up of crystalline deposits on the stent surface, making stent removal difficult and painful. This hypothesis was confirmed and extended by this study.

Performed EDS study (Table 1) showed that the inner and outer surfaces of the ureteral catheter were covered and deposited with the crystals of urea and, more importantly, also by the post URS-L fragments. Such a distribution of film components suggests that the blockage of the ureteral stent is due to not only large kidney stones but also to a continuous flow of small calculi growing on one another.

**Table 1:** The results of EDS analysis of outer, proximal part of DJ ureteral stent, implanted in children body during 13 days

EDS Spectrum	Element	Weight [%]
	C	54.15 ± 2.34
	O	19.87 ± 1.74
	Na	10.80 ± 1.23
	Cl	9.63 ± 1.88
	Ca	4.56 ± 0.52

**Fig. 6:**
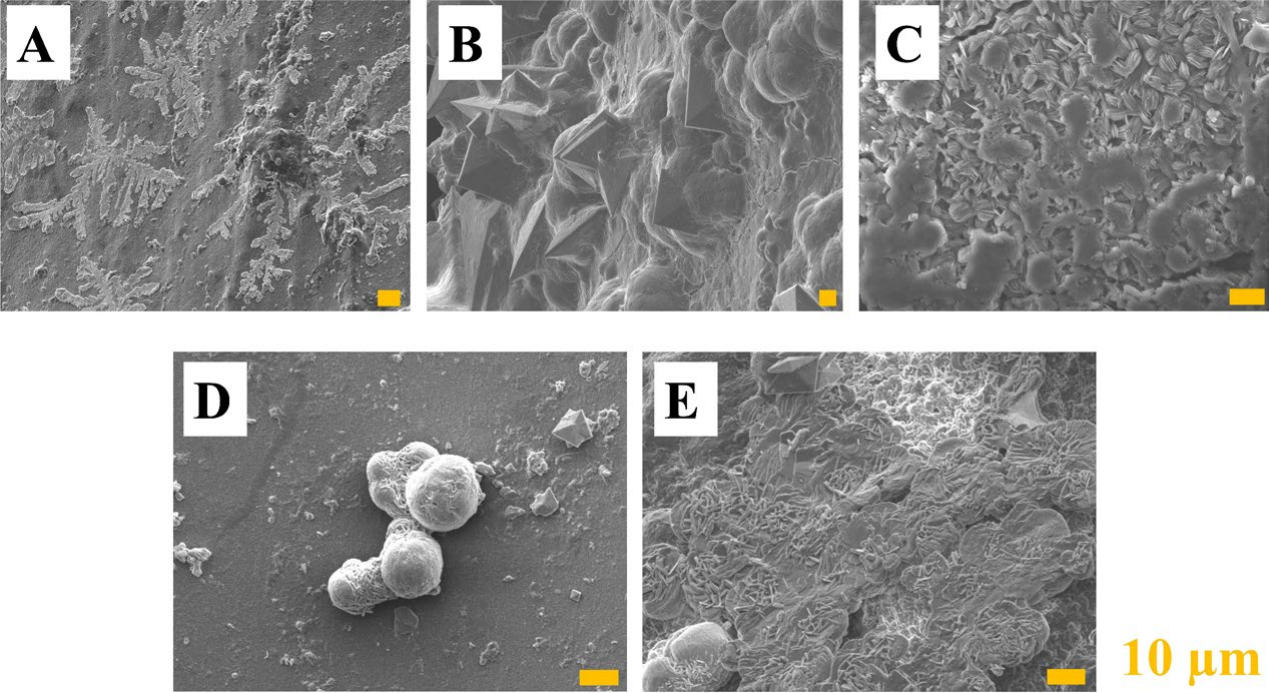
SEM micrographs of ureteral stent encrustations and biofilms, where: **a** NaCl dendrimer; **b** NaCl crystals and calcium carbonate particles, **c** calcium oxalate dihydrate, **d** *S. anginosus* or *A. tetradius*, and **e** *L. jensenii*

**Fig. 7:**
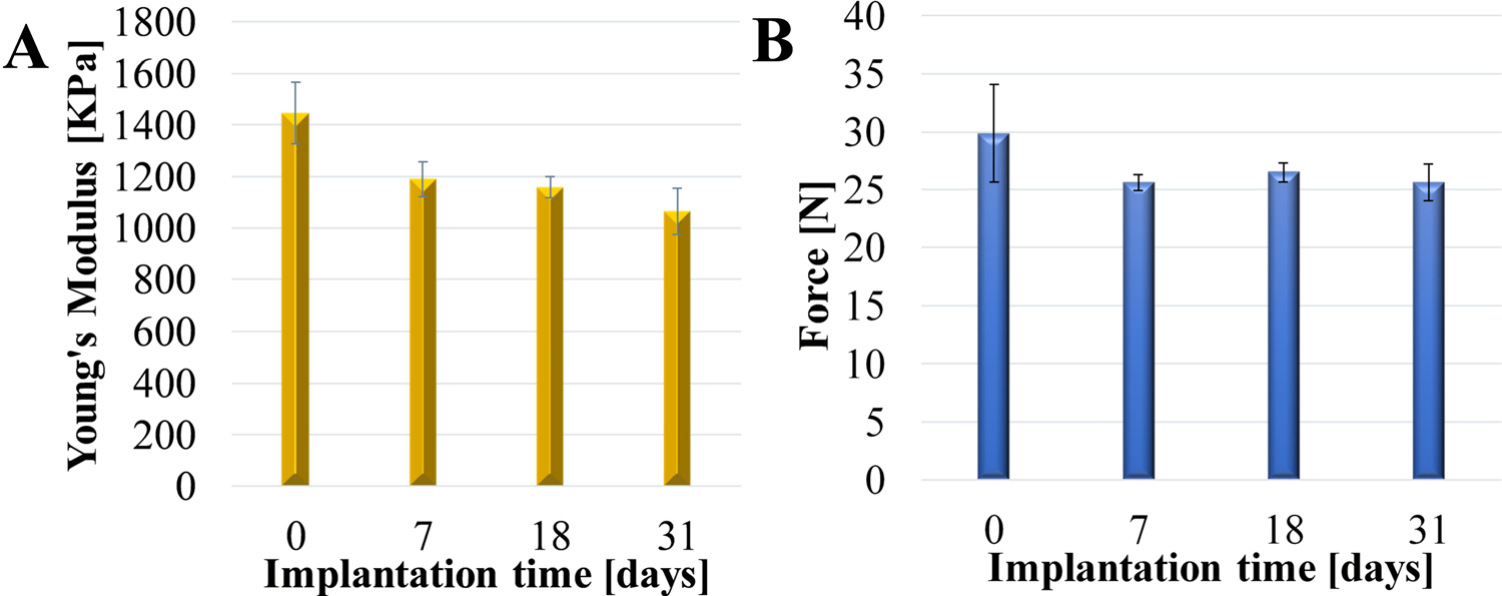
The effects of implantation time in vitro on the Young’s modulus (**a**), and ultimate force (**b**) of styrene/ethylene/butylene block copolymer ureteral stents (containing side-drainage holes)

Additionally, a performed analysis showed that the region of ureteral stents most susceptible to crystal, calculi, and biofilm deposition was distal and proximal parts (Figs. 2, 3, 4 and 5). This is extremely dangerous due to the risk of biofilm formation in this region as well as urinary infection. Performed results indicate the necessity to use antibacterial and anticoagulation coatings in the distal and proximal pigtail of DJ stents [35]. Additional in-depth microscopic analysis of ureteral stents after implantation (Fig. 7) confirmed the above-mentioned conclusions. The surface of the DJ stents made of silicone-modified styrene/ethylene/butylene block copolymer was covered with a more complex multi-layer consisting mostly of dentritic (Fig. 7a) and precipitated crystals of NaCl (Fig. 6b), post-URS-L fragments (calcium oxalate stone in a tetragonal dipyramidal shape Fig. 6c). The bulk of the encrustations appeared to consist of crystals or fibrous organic deposits (Fig. 7a–c). Imaging revealed that only a few samples exhibited visible bacteria, likely to include *Streptococcus anginosus* or *Anaerococcus tetradius* (Fig. 7d) and *Lactobacillus jensenii* (Fig. 7e) [33].

Prevention of stone-crystal-layer formation on DJ stents is also extremely important due to the resultant increased pressure in the blocked stent, causing a reflux (retrograde flow of urine from the bladder to the kidney), which may result in infections, scarring, and even renal failure [38]. Moreover, the build-up of deposits on the stent surface influences the stent’s mechanical strength, which results in increasing stiffness and greater susceptibility to cracks and fracture. The mechanical properties of unused stents and stents retrieved from patients following insertion from 7 to 31 days, all containing side-drainage holes are presented in Fig. 7. The Young’s modulus (*E*) was calculated for each DJ stent using the engineering stress, which assumes no changes in the cross-sectional area. The DJ stent before implantation was the stiffest (*E* = 1446±120 kPa), and the DJ stent implanted for 31 days was the least stiff (*E* = 1064±90 kPa). These results are consistent with several studies to date [26, 39, 40]. Whereas highly elastic and flexible materials may be more likely to kink or bend in vivo, more rigid materials could cause severe bladder urgency and haematuria or fracture during long-term stenting [41]. In general, there was no significant change in ultimate force of DJ stents following implantation during 7 days in comparison to 31 days. Increased implantation time caused an increase in Young’s modulus, indicating that decreased stent rigidity had occurred.

## Conclusion

Analysis of surface morphologies of double-J ureteral stents implanted after utilizing ureterorenoscopic-lithotripsy procedure to treat calcium oxalate for 7–31 days indicated that crystalline and calculi deposition was observed even 7 days after implantation in children patients. The stone-crystal layer was formed both on the outer and inner sides of the ureteral stents. However, the distal and proximal parts of stent are the most susceptible for post URS-L fragments and urea salt deposition. Residues deposited on the surface form a homogeneous multi-layer, which close the lumen of the DJ stents and significantly affect the flow of urine. Mechanical tests indicate that insertion of DJ stents results in slight decrease of Young’s modulus; however, it cannot be the reason of ureteral stent cracking. The surfaces of the ureteral stents need improvement to minimize salt and kidney stone deposition, causing pre-biofilm formation, as well as the occurrence of defects and cracks. Performed analysis confirms the bacteria adsorption on ureteral stent and its contribution to the encrustations, despite the low bacterial load in urinary tract sample. Performed analysis pointed to the need to use an antibacterial coating in the above-mentioned part of the ureteral stent to prolong its usage time and to prevent urinary tract infection.

## References

[1] Aleling T, Petros B (2018). Kidney stone disease: an update on current concepts. Adv Urol.

[2] Schwaderer AL, Raina R, Anshika K, Fayez S, Sharon MM, Kusumi K (2019). Comparison of risk factors for pediatric kidney stone formation: the effects of sex. Front Pediatr.

[3] Milicevic S, Bijelic R, Jakovljevic B (2015). Encrustation of the ureteral double J stent in patients with a solitary functional kidney—a case report. Med Arch.

[4] Halinski A, Halinski A, Zaniew M, Kudliński B, Soltysiak J, Sobolewski B, Steyaert H (2019). Interest of URS-L in the treatment of ureterolithiasis in preschool children. Front Pediatr.

[5] Lawrence EL, Turner IG (2005). Materials for urinary catheters: a review of their history and development in the UK. Med Eng Phys.

[6] Yin K, Divakar P, Wegst UG (2019). Freeze-casting porous chitosan ureteral stents for improved drainage. Acta Biomater.

[7] Singha P, Locklin J, Handa H (2017). A review of the recent advances in antimicrobial coatings for urinary catheters. Acta Biomater.

[8] Tunney M, Gorman S (2002). Evaluation of a poly(vinyl pyrollidone)-coated biomaterial for urological use. Biomaterials.

[9] Tunney MM, Keane PF, Jones DS, Gorman SP (1996). Comparative assessment of ureteral stent biomaterial encrustation. Biomaterials.

[10] Roupret M, Daudon M, Hupertan V (2005). Can ureteral stent encrustation analysis predict urinary stone composition?. Urology.

[11] Scarneciu I (2018). The risk factors and chemical composition of encrustation of ureteral double J stents in patients with urolithiasis. Rev Chim.

[12] Flores-Mireles AL, Walker JN, Caparon M, Hultgren SJ (2015). Urinary tract infections: epidemiology, mechanisms of infection and treatment options. Nat Rev Microbiol.

[13] Shabeen AKS, Bhargava R, Manzoor MAP, Mujeeburahiman M (2018). Characteristics of bacterial colonization after indwelling double-J ureteral stents for different time duration. Urol Ann.

[14] Nycz M, Paradowska E, Arkusz K, Kudlinski B, Krasicka-Cydzik E (2018). Surface analysis of long-term hemodialysis catheters made of carbothane (poly(carbonate)urethane) before and after implantation in the patients’ bodies. Acta Bioeng Biomech.

[15] Paradowska E, Nycz M, Arkusz K, Kudlinski B, Krasicka-Cydzik E, Arkusz K, Bedzinski R, Klekiel T, Piszczatowski S (2019). Impedimetric method to monitor biological layer formation on central venous catheters for hemodialysis made of carbothane. Biomechanics in medicine and biology. BIOMECHANICS, (2018) advances in intelligent systems and computing.

[16] Jacobsen SM, Shirtliff ME (2011). Proteus mirabilisbiofilms and catheter-associated urinary tract infections. Virulence.

[17] Arkusz K, Krasicka-Cydzik E (2018). The effect of phosphates and fluorides, included in TiO2 nanotube layers, on the performance of hydrogen peroxide detection. Arch Metall Mater.

[18] Jacobsen SM, Stickler DJ, Mobley HLT, Shirtliff ME (2008). Complicated catheter-associated urinary tract infections due to Escherichia coli and Proteus mirabilis. Clin Microbiol Rev.

[19] Lo J, Lange D, Chew B (2014). Ureteral stents and foley catheters-associated urinary tract infections: the role of coatings and materials in infection prevention. Antibiotics.

[20] Nycz M, Arkusz K, Pijanowska DG (2019). Influence of the silver nanoparticles (AgNPs) formation conditions onto titanium dioxide (TiO2) nanotubes based electrodes on their impedimetric response. Nanomaterials.

[21] Paradowska E, Arkusz K, Pijanowska DG (2019). The influence of the parameters of a gold nanoparticle deposition method on titanium dioxide nanotubes, their electrochemical response, and protein adsorption. Biosensors.

[22] Kotaskova I, Obrucova H, Malisova B, Videnska P, Zwinsova B, Peroutkova T, Freiberger T (2019). Molecular techniques complement culture-based assessment of bacteria composition in mixed biofilms of urinary tract catheter-related samples. Front Microbiol.

[23] Zelichenko G, Steinberg D, Lorber G, Friedman M, Zaks B, Lavy E, Duvdevani M (2013). Prevention of initial biofilm formation on ureteral stents using a sustained releasing varnish containing chlorhexidine. Vitro study. J Endourol.

[24] Diaz Blanco C, Ortner A, Dimitrov R, Navarro A, Mendoza E, Tzanov T (2014). Building an antifouling zwitterionic coating on urinary catheters using an enzymatically triggered bottom-up approach. ACS Appl Mater Interfaces.

[25] Fisher LE, Hook AL, Ashraf W, Yousef A, Barrett DA, Scurr DJ, Bayston R (2015). Biomaterial modification of urinary catheters with antimicrobials to give long-term broadspectrum antibiofilm activity. J Control Release.

[26] Hendlin K, Dockendorf K, Horn C, Pshon N, Lund B, Monga M (2006). Ureteral stents: coil strength and durometer. Urology.

[27] Joshi HB, Chitale SV, Nagarajan M, Irving SO, Browning AJ, Biyani CS (2005). A prospective randomized single-blind comparison of ureteral stents composed of firm and soft polymer. J Urol.

[28] Venkatesan N, Shroff S, Jayachandran K, Doble M (2010). Polymers as ureteral stents. J Endourol.

[29] Beiko DT, Knudsen BE, Denstedt JD (2003). Reviews in endourology-advances in ureteral stent design. J Endourol.

[30] Kim KW, Kim HH, Choi YH, Lee SB, Baba Y (2020). Urine flow analysis using double J stents of various sizes in in vitro ureter models. Int J Numer Methods Biomed Eng.

[31] Nestler S, Witte B, Schilchegger L (2019). Size does matter: ureteral stents with a smaller diameter show advantages regarding urinary symptoms, pain levels and general health. World J Urol.

[32] Mosayyebi A, Manes C, Carugo D (2018). Advances in ureteral stent design and materials. Curr Urol Rep.

[33] Buhmann MT, Abt D, Nolte O, Neu TR, Strempel S, Albrich WC, Betschart P, Zumstein V, Neels A, Maniura-Weber K, Ren Q (2019). Encrustations on ureteral stents from patients without urinary tract infection reveal distinct urotypes and a low bacterial load. Microbiome.

[34] Dutta Choudhury M, Dutta T, Tarafdar S (2015). Growth kinetics of NaCl crystals in a drying drop of gelatin: transition from faceted to dendritic growth. Soft Matter.

[35] Mosayyebi A, Vijayakumar A, Yue QY, Bres-Niewada E, Manes C, Carugo D, Somani BK (2017). Engineering solutions to ureteral stents: material, coating and design. Cent Eur J Urol.

[36] Sali GM, Joshi HB (2019). Ureteric stents: overview of current clinical applications and economic implications. Int J Urol.

[37] Lojanapiwat B, Muttarak M (2004). Noninvasive assessment of patency of internal ureteral stent: role of colour doppler ultrasound. Asian J Surg.

[38] Cummings LJ, Waters SL, Wattis JAD, Graham SJ (2004). The effect of ureteric stents on urine flow: reflux. J Math Biol.

[39] Pedro RN, Hendlin K, Kriedberg C, Monga M (2007). Wire-based ureteral stents: impact on tensile strength and compression. Urology.

[40] Gorman SP, Jones DS, Bonner MC, Akay M, Keane PF (1997). Mechanical performance of polyurethane ureteral stents in vitro and ex vivo. Biomaterials.

[41] Tomaszewski M, Sybilski K, Baranowski P, Malachowski J (2020). Experimental and numerical flow analysis through arteries with stent using particle image velocimetry and computational fluid dynamics method. Biocybern Biomed Eng.

